# Towards Advances in Medicinal Plant Antimicrobial Activity: A Review Study on Challenges and Future Perspectives

**DOI:** 10.3390/microorganisms9102041

**Published:** 2021-09-27

**Authors:** Natalia Vaou, Elisavet Stavropoulou, Chrysa Voidarou, Christina Tsigalou, Eugenia Bezirtzoglou

**Affiliations:** 1 Laboratory of Hygiene and Environmental Protection, Department of Medicine, Democritus University of Thrace, Dragana, 68100 Alexandroupolis, Greece; empezirt@med.duth.gr; 2 Department of Infectious Diseases, Centre Hospitalier Universitaire Vaudois (CHUV), Rue du Bugnon, 1011 Lausanne, Switzerland; 3 Department of Agriculture, University of Ioannina, 47132 Arta, Greece; xvoidarou@uoi.gr; 4 Laboratory of Microbiology, Department of Medicine, Democritus University of Thrace, Dragana, 68100 Alexandroupolis, Greece; xtsigalou@yahoo.gr

**Keywords:** medicinal plants, bioactive compounds, antimicrobial activity, new antimicrobials, challenges, future perspectives

## Abstract

The increasing incidence of drug- resistant pathogens raises an urgent need to identify and isolate new bioactive compounds from medicinal plants using standardized modern analytical procedures. Medicinal plant-derived compounds could provide novel straightforward approaches against pathogenic bacteria. This review explores the antimicrobial activity of plant-derived components, their possible mechanisms of action, as well as their chemical potential. The focus is put on the current challenges and future perspectives surrounding medicinal plants antimicrobial activity. There are some inherent challenges regarding medicinal plant extracts and their antimicrobial efficacy. Appropriate and optimized extraction methodology plant species dependent leads to upgraded and selective extracted compounds. Antimicrobial susceptibility tests for the determination of the antimicrobial activity of plant extracts may show variations in obtained results. Moreover, there are several difficulties and problems that need to be overcome for the development of new antimicrobials from plant extracts, while efforts have been made to enhance the antimicrobial activity of chemical compounds. Research on the mechanisms of action, interplay with other substances, and the pharmacokinetic and/or pharmacodynamic profile of the medicinal plant extracts should be given high priority to characterize them as potential antimicrobial agents.

## 1. Introduction

The World Health Organization (WHO) has stated that 80% of the developing world still benefits from the use of traditional medicines derived from medicinal plants [1,2,3]. The total estimated number of plants is approximately 374,000 [4] in comparison to 28,187 medicinal species used by humans [5]. WHO has also recorded the names of over 20,000 species of medicinal plants [6] and described medicinal plants as one of the potential sources of new drugs [7]. More than 100 countries have developed regulations for medicinal plants. There are over 1340 plants with defined antimicrobial activity and over 30,000 antimicrobial compounds have been isolated from plants [8]. Moreover, it has been estimated that 14–28% of higher plant species are medicinal and that 74% of bioactive plant-derived compounds were discovered based on ethnomedicinal uses [9].

The extensive, inappropriate, irregular, and indiscriminate uses of antibiotics have resulted in the emergence of antimicrobial resistance, making many currently available medications ineffective [10,11,12]. This emerging trend is concerning and considered by the WHO to be perhaps the most urgent issue facing medical science [13]. Therefore, there is an increasing demand to develop new antimicrobial agents that are able to decrease the use of antibiotics and to face resistance development. This has directed researchers to isolate and identify new bioactive chemicals from plants to act against microbial resistance [14,15,16,17], also considering that approximately 50% of current pharmaceuticals and nutraceuticals are natural products and their derivatives [18]. Medicinal plants yield an almost unlimited source of bioactive compounds and their use as antimicrobial agents has been exploited in different ways [19,20]. Notwithstanding, the compounds have not yet been thoroughly investigated [21]. Natural antimicrobial agents can act alone or in combination with antibiotics to enhance antimicrobial activity against a wide range of microbes [22,23]. As the antimicrobial action of many medicinal plants is still unexplored, researchers are increasingly targeting the search for fast-growing new and effective treatments. [24,25].

The information required to evaluate the efficacy of potentially important medicinal plants and to prove their antimicrobial worth needs to be efficient and well-validated. Therefore, to obtain a more comprehensive perspective of the potential use of medicinal plant extracts as alternative solutions to combat drug resistance, the most relevant studies regarding the validation of the antimicrobial activity of medicinal plants, the underlying mechanisms of action, the mechanisms of bacterial resistance, the plant-derived chemical compounds that may be responsible for such activity, the challenges and future perspectives of medicinal plant antimicrobial activity were critically analyzed in this review.

## 2. Antimicrobial Activity of Medicinal Plant Extracts

Extracts isolated from medicinal plants have been reported exhibit various biological activities such as antimicrobial, anti-inflammatory, and antioxidant activities [26]. The antimicrobial compounds from medicinal plants may inhibit the growth of bacteria, fungi, viruses, and protozoa by different mechanisms than those of presently used antimicrobials and may have a significant clinical value in the treatment of resistant microbial strains [27]. Some of those active compounds show both intrinsic antibacterial activity and antibiotic resistance-modifying activities and some of them, while not effective as antibiotics by themselves, when combined with antibiotics, can help overcome antibiotic resistance in bacteria. Chemically complex compounds have great therapeutic potential as they have fewer side effects compared to synthetic drugs and also low chances of developing resistance [28,29,30]. Bacteria may develop resistance to medicinal plants treatment if only one active ingredient with a specific target is involved, a condition similar to an antibiotic [31]. However, since the literature on bacteria developing resistance plants is limited then further research on resistance mechanisms is required [32]. Furthermore, the effectiveness of medicinal plant extracts to inhibit bacteria growth is also related to the synergistic effect between the active compounds of the extracts [33]. The synergism action come from different effects, namely the emergence of multi-target mechanisms, the existence of compounds capable of suppressing bacterial resistance mechanisms, pharmacokinetic or physicochemical effects resulting in enhanced bioavailability, solubility and resorption rate, neutralization of adverse effects and reduction of toxicity [33].

Medicinal plants are rich in a wide variety of chemical compounds, which have been found in vitro to have antimicrobial activities [28,34]. It is extremely difficult to include all the medicinal plants and their compounds of potent antimicrobial activity in this review. However, some compounds of high interest are being presented below.

Phytochemical studies identified the presence of different compounds such as spermidine, rutin, quercetin, tocopherol, and carotenoids, derived from caper (*Capparis* sp.) responsible for antimicrobial, antioxidative, anti-inflammatory, and antiviral activities. Seed extracts of *Capparis decidua* showed antibacterial, antifungal, and antileishmanial activity probably due to quaternary ammonium and glucosinolate [35]. The use of bearberry (*Arctostaphylos ura-ursi*) and cranberry juice (*Vaccinium macrocarpon*) to treat urinary tract infections have been published, while species such as lemon balm (*Melissa officinalis*), garlic (*Allium sativum*), and tea tree (*Melaleuca alternifolia)* are described as broad-spectrum antimicrobial agents [36]. Phenolics, alkaloids, flavonoids, triterpenes, and steroids from Cameroonian plants were the most bioactive compounds revealing significant antimicrobial activity [37,38]. The the active indredient of Fulyzaq (crofelemer, a proanthocyanidin oligomer0, was isolated from the plant *Croton lechleri* (*Euphorbiaceae*) found in the Western Amazonian regions of South America [39]. The leaf extracts of *Myrtus communis* and *Verbena officinalis* exhibited good antibacterial activity against *Staphylococcus aureus*, *Escherichia coli*, and *Salmonella typhi*. *Myrtus communis* also displayed remarkable activity against *Pseudomonas aeruginosa*. Carrot (*Daucus carota*) seed oil, and tea tree (*Melaleuca alternifolia*) oil show antimicrobial activity against *Helicobacter pylori* and *Mycoplasma pneumoniae*, respectively [40]. Methanol extracts of *Oxalis corniculata*, *Artemisia vulgaris*, *Cinnamomum tamala*, and *Ageratina adenophora* exhibited antimicrobial activities against *Escherichia coli*, *Salmonela Typhi*, *Klebsiella pneumoniae*, *Staphylococcus aureus*, and *Citrobacter koseri* [41] Also, hydromethanolic extracts of *Berberis vulgaris*, *Cistus monspeliensis*, and *Punica granatum* demonstrated high activity against *Staphylococcus aureus*, *Enterococcus faecalis*, and *Enterobacter cloacae* [42].

An endophytic fungus isolated from the medicinal plant *Hypericum acmosepalum* contained some compounds including hyperenone A, hypercalin B, and hyperphorin and emodin, responsible for antibacterial activity on resistant *Staphylococcus aureus*, on *Klebsiella pneumoniae*, *Pseudomonas aeruginosa*, *Salmonella enterica*, *Escherichia coli*, *Mycobacterium tuberculosis*, upon the fungal strains *Aspergillus niger* and *Candida albicans* [43]. The *Hypericum olympicum* contains numerous essential oil compounds, with the main components being *E*-anethole, β-farnesene and spathulenol, while other components included *E*-caryophyllene, germacrene D, terpenes and new type of acylphloroglucinol. The crude methanol extract of Hypericum olympicum showed a broad spectrum of very strong antimicrobial activity, with the highest activity observed against Klebsiella pneumoniae and Salmonella enteritidis [44]. Natural resins derived mostly from medicinal plants and their compounds revealed antibacterial and antiprotozoal activity [45,46]. In particular, the extract of propolis richer in flavonoids (pinocembrin and galangin) was more active against *Streptococcus pyogenes* strains [47]. The antimicrobial effect of Korean propolis was studied against *Streptococcus mutans* [48]. The compound diaporthalasin yielded from the fungus *Diaporthaceae* sp. from a marine sponge displayed potent antibacterial activity against both *Staphylococcus aureus* and methicillin-resistant *Staphylococcus aureus* (MRSA) [49]. Essential oils derived from aromatic medicinal plants, like fennel, peppermint, thyme, lavender, and containing mixtures of volatile substances, such as monoterpenes, sesquiterpenes, and phenylpropanoids, have been reported to be active on Gram-positive and Gram-negative bacteria and on fungi and viruses [50,51].

## 3. Mechanisms of Action of Antimicrobial Agents

**Figure 1:**
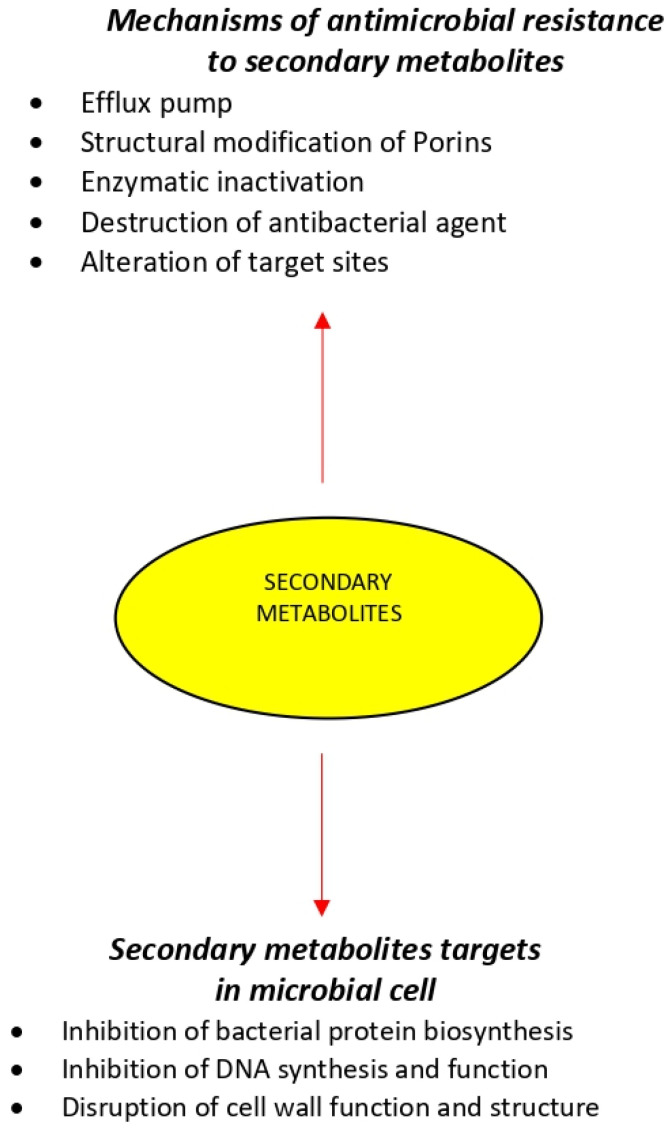
Mechanisms of antimicrobial agents and resistance by pathogens.

The antimicrobial activity of an agent is mainly attributed to two mechanisms, which include interfering chemically with the synthesis or function of vital components of bacteria and/or circumventing the conventional mechanisms of antibacterial resistance. However, bacteria can create resistance to multiple antimicrobials inherently by selective pressures or acquire the resistance machinery from neighboring microbe [52,53]. The mechanism described below correspond to known antimicrobial drugs (Figure 1).

### 3.1. Bacterial Protein Biosynthesis

The inhibition of protein synthesis by targeting the bacterial ribosomal subunits is an effective approach to combat bacterial infections. Antibiotics like macrolides, tetracyclines, aminoglycosides, and oxazolidinones show antibacterial activities through this mechanism. Amikacin binds permanently to 16 S rRNA and the RNA-binding S12 protein of the prokaryotic ribosome’s 30 S subunit, inhibiting protein synthesis by changing the ribosome’s shape so that it cannot read the mRNA codon correctly. It also interferes with the part that interacts with the wobbling base of the tRNA anticodon [54].

### 3.2. Inhibition of Nucleic Acid Synthesis

DNA gyrase is known as the enzyme that is essential for the synthesis, replication, repair, and transcription procedures of bacterial DNA. Therefore, gyrase can be considered a fine target for antibacterial agents including nalidixic acid, as well as for fluoroquinolones, such as ciprofloxacin. Ciprofloxacin functions by inhibiting a type II topoisomerase (DNA gyrase) and topoisomerase IV, necessary to separate bacterial DNA, thereby inhibiting cell division [55].

### 3.3. Cell-Wall Biosynthesis

Bifunctional enzymes—transglucosilases and transpeptidases—that play critical roles in the formation of the bacterial cell wall are suitable targets for bactericidal antibiotics including penicillin, cephalosporins, and vancomycin. These antibiotics can bind the peptide substrate of the peptidoglycan layer and thus prevent an enzyme reaction. Vancomycin acts by inhibiting proper cell wall synthesis in Gram-positive bacteria.The large hydrophilic molecule of Vancomycin can form hydrogen bond interactions with the N-acatylmuramic acid (NAM)/N-acetylglucosamine (NAG) peptides’s terminal D-alanyl-D-alanine moieties. Vancomycin binds to the D-Ala-D-Ala and prevents the formation of the long polymers of NAM and NAG that form the cell wall’s backbone strands [56].

### 3.4. Destruction of Bacterial Cell Wall

Various antibiotics, like polymyxins, can bind to the lipid component of lipopolysaccharide and thus cause structural alterations by the means of phospholipid exchange that might end in an osmotic imbalance and finally rapid bacterial death. Polymyxin B, alters bacterial outer membrane permeability by binding to a negatively charged site in the lipopolysaccharide layer, that has an electrostatic attraction for the positively charged amino groups in the cyclic peptide portion; the result is destabilized outer membrane. Moreover, the fatty acid component dissolves in hydrophobic region of cytoplasmic membrane and disrupts membrane integrity. This causes leakage of cellular molecule and inhibition of cellular respiration [57].

## 4. Mechanisms of Resistance to Antimicrobial Agents

Extensive use and misuse of antibiotics have led to the emergence of multidrug resistance (MDR) in a variety of pathogenic bacteria [58,59]. Antimicrobial resistance is a complex global public health challenge and is generally due to resistance genes and their downstream effects. These traits can be inherited, imported from other pathogens, or may occur through random mutations in bacterial DNA [60]. No single or simple strategy will suffice to fully contain the emergence and spread of infection organisms that become resistant to the available antimicrobial drugs [61,62]. The current shortage of new antimicrobials to replace those that become ineffective brings an urgent need to maintain the effectiveness of existing drugs [10]. Bacteria can show resistance to antibacterial agents through a variety of mechanisms, which are discussed separately below (Figure 1).

### 4.1. Efflux Pump

Throughout the mechanism of the efflux pump (EP) the antibacterial agent is pumped out faster than the time it requires to be diffused in bacterial cells and consequently, the intrabacterial concentration becomes much lower than the effective value. By reducing the intrabacterial concentration of EP-mediated inhibitors of protein synthesis systems such as ribosomes, bacterial protein synthesis procedures are often performed without interruption [63]. Antibiotic resistance via the mechanism of EPs can be observed in a wide range of pathogenic Gram-positive and Gram-negative bacteria and fungi such as *Staphylococcus aureus*, *Pseudomonas aeruginosa*, *Acinetobacter baumannii* and *Candida albicans* [64]. Specifically, in Gram-negative bacteria, the effect of EPs in combination with reduced drug uptake due to the multi-membranar layer is responsible for the high intrinsic and acquired antibiotic resistance often associated with this group of microorganisms [65]. Therefore, employing EP inhibitors in combination with antibacterial agents is often contemplated as an efficient approach for the aim of combating microbial infections.

### 4.2. Structural Modification of Porins

Antibiotics influx is mainly controlled by porins which are proteins able to form water-filled open channels that allowing the passive transportation of molecules across lipid bilayer membranes [66]. Variation in porin structure results in alteration of membrane permeability and is a mechanism to escape from the antibacterial agents [67]. This type of antibacterial resistance is frequently found in Gram-negative pathogens such as *Acinetobacter* spp. and *Pseudomonas* spp. [66].

### 4.3. Enzymatic Inactivation

Resistance to aminoglycosides in Gram-negative bacteria is most often mediated through the modification of functional groups by utilizing three kinds of modifying enzymes. These modified products have displayed a considerably lower affinity for RNA and have caused the blockage of protein synthesis since they are not capable of binding to ribosomes [68,69].

### 4.4. Destruction of Antibacterial Agent

Another strategy of bacterial resistance is the chemical decomposition of antibiotics or antibacterial agents by changing the chemical type. Degradation is mediated by the binding of the hydrolytic enzyme, β-lactamase, to the β-lactam ring of penicillin, cephalosporins, and carbapenems [70,71].

### 4.5. Alteration of Target Sites

Drug-binding site alteration can be counted as another resistance mechanism, in which the antibacterial agent is not able to react with the targeted bacterial site and thus results in a dramatic reduction in the antibacterial activity of the agent [72]. An example of this mechanism is vancomycin resistance in vancomycin-resistant enterococci species in which van HAX genes encode a new pathway of enzymes that induce structural modifications by switching from the amide linkage in the D-Ala-D-Ala peptidoglycan structure to the ester linkage in the D-Ala-D-Lac structure resulting in reduced the antibiotic-binding affinity [73].

## 5. Antimicrobial Activity Mechanisms of Medicinal Plant-Derived Chemical Compounds

Although synthetic antimicrobial agents are already approved in many countries, the usage of medicinal plant-derived natural compounds continues to attract the attention of many researchers [74]. Medicinal plants have enormous potential for the discovery of new bioactive compounds which can fight against resistant microorganisms [75,76]. Medicinal plant-derived chemicals are a wide group of chemical compounds that have been found naturally in plants. They can restore the clinical application of older antibiotics by increasing their potency and therefore, avoid the fact of resistance [28,77].

Plant-derived bioactive compounds (phytochemicals) of therapeutic value are mostly secondary metabolites used for medicinal purposes. Secondary metabolites are the results of secondary plant metabolism and can occur as intermediate or end products [78]. They have a wide antimicrobial activity range according to the structure, number, and position of substituent groups, presence of glycosidic linkages alkylation of OH groups, and the topography and climate of the country of origin. Indeed, variations in the quality and quantity of bioactive secondary metabolites modify their antimicrobial activity against different microbial strains [79,80,81].

In most cases, bioactive plant extracts contain complex mixtures of ingredients, and their synergistic action can yield an enhanced effect [34]. The microbial cell can be affected by these compounds in several ways. In general, bioactive compounds primary target site is the cytoplasmic membrane, affecting its structure and integrity, permeability, or functionality in different ways [25,82,83]. It has been suggested that plant extracts may contain inhibitors of EP in their composition [25]. In addition, inhibition of normal cell communication [quorum sensing (QS)] has been also described as one of the most promising mechanisms of action of bioactive compounds against MDR pathogens. QS inhibitors should have the ability to decrease the expression of QS-controlled genes and being chemically stable to resist the metabolic and disposal processes of the host organism [25,82]. Certain compounds can modify or inhibit the protein-protein interactions, thus presenting themselves as effective modulators of immune response, mitosis, and apoptosis [84]. Moreover, they have the ability to interfere with intermediary metabolism [85], to induce the coagulation of cytoplasmic constituents [86] and disrupt or inhibit the formation of biofilms, which confer a protective advantage to pathogens during infection [87,88]. The presence of multiple antiviral components in medicinal plant extracts interfaces with different viral proteins at various stages of viral replication [89].

Although there is an extensive existence of these compounds, based on their chemical structures, chemical composition, biosynthetic pathway, or their solubility, they can be classified into several main groups that include alkaloids, phenolic compounds, sulfur-containing compounds, coumarins, terpenes/essential oils, and lectins and polypeptides [90,91]. The mechanisms of action, and antimicrobial activity of the most important compounds from those chemical groups are described below and listed in Table 1.

### 5.1. Alkaloids

Alkaloids are chemically very diverse structures of heterocyclic nitrogen compounds characterized by analgetic, antispasmodic, and antimicrobial effects [114]. In particular, many studies have indicated that these compounds are commonly found to play a significant role in the treatment of many infections [115,116]. Activity against Gram-negative bacteria and yeast was displayed by indoquinoline alkaloids [117] while the alkaloid quinine is popular for its antiprotozoal activity against the malarial parasite [118]. Most of the alkaloids act through EP inhibitory activity. Berberine, an isoquinoline alkaloid, accumulates in cells driven by the membrane potential and is an excellent DNA intercalator active in several microorganisms and a target on RNA polymerase, gyrase, and topoisomerase IV and on nucleic acid [119,120]. Thus, berberine disrupts the membrane structure by increasing the membrane permeability of bacteria [121].

### 5.2. Phenolic Compounds/Polyphenols

Phenolic compounds are one of the most diverse groups of bioactive secondary metabolites found in medicinal plants. They are extensively utilized against pathogenic bacteria [79,83,122,123]. However, their activity is generally weak and is often non-specific [124]. Phenolic compounds include flavones, flavanols, flavonoids, quinones, and tannins [7,123]. These compounds showed diverse mechanisms of action against different microbial strains. The known mechanisms include the EP inhibitory activity, the capability for modifying cell membranes permeability, the shifting in several intracellular functions caused by the phenolic compounds to enzymes binding, or by the loss of the cell wall integrity due to various interactions with the cell membrane [125,126,127,128,129,130]. In particular, flavones represent an antimicrobial agent to disrupt microbial envelopes [131]. Flavanols form complexes with the microbial cell wall and inactivate specific microbial enzymes, possibly through reaction with sulfhydryl groups or through more non-specific interactions with the proteins [125,126,132]. Flavonoids are phenolic compounds that are well known for their antimicrobial, antiviral, and anti-inflammatory properties [133]. Some flavonoids showed a promising activity on *Escherichia coli* and *Pseudomonas aeruginosa* an revealed activity against *Klebsiella pneumoniae* and *Mycobacterium tuberculosis* [134,135]. The antimicrobial properties of flavonoids are thought to come from the power to form complexes with both extracellular proteins, as well as with bacterial membranes. Therefore, their antimicrobial activity is through inhibition of bacterial virulence factors such as QS signal receptors and enzymes, destabilization and permeabilization of the cytoplasmic membrane, inhibition of extracellular microbial enzymes, and deprivation of the substrates required for microbial growth such as iron and zinc [34,136]. In addition to providing a source of stable free radicals, quinones are known to complex with nucleophilic amino acids in microbial proteins often leading to loss of their action [135]. Tannins are characterized by antibacterial activity against both Gram-negative and Gram-positive bacteria, occurring through mechanisms including disruption of cell wall and membrane, inhibition of oxidative phosphorylation which affects microbial metabolism, intercalation into DNA base pairs, and inhibition of microbial enzymes which generally affect transcription, repress expression, and cause cell death [137].

### 5.3. Sulfur-Containing Compounds

There is extensive literature on the topic of antibacterial, antifungal, antiviral, and antiprotozoal activities of sulfur-containing compounds that are obtained from plants with high concentrations of polysulphides [138,139,140,141]. The most important compounds are allicin, ajoene, and isothiocyanates. These compounds have been detected to be effective against both Gram-positive and Gram-negative bacteria including *Helicobacter pylori* [77,142,143,144,145,146]. Antimicrobial mechanisms of sulfur-containing compounds may include the inhibition of sulfhydryl-dependent enzymes and partial inhibition of the DNA and protein synthesis as well [147,148]. Some compounds can also damage the cell wall integrity and lead to the leakage of cellular metabolites [149,150]. Antifungal activities of these compounds might be related to decreased rate of oxygen consumption, intracellular accumulation of reactive oxygen species, and the depolarization of mitochondrial membrane [151].

### 5.4. Coumarins

Coumarins are phenolic substances with antimicrobial activity of both their normal and synthetic derivatives [134,152,153]. Particularly, coumarins extracts from many medicinal plants are active against strains of *Salmonella enterica Typhi*, *Enterobacter aerogenes*, *Enterobacter cloacae*, *Bacillus subtilis*, *Klebsiella pneumoniae*, *Staphylococcus aureus*, MRSA, and *Helicobacter pylori* [105,106]. Coumarins can suppress the QS network of bacterial pathogens, i.e., the production of small signal molecules by bacterial cells and affect their ability in the development of biofilm formation and virulence factor production [154,155]. Moreover, some coumarins show inhibition against the EP system in MRSA [108] and are were also potent inhibitors of DNA gyrase [107]. Coumarins have also been found to stimulate macrophages, which could have an indirect negative effect on infection [34].

### 5.5. Terpenes

Terpenes are also referred to as isoprenoids and their derivatives that contain additional elements, usually oxygen, are called terpenoids. They are considered the most diverse family of natural products that perform numerous functions ranging from participation in the primary structure of cells to contribution to the cell functions [156,157]. Terpenes are the main component of essential oils fractions, which carry the peculiar fragrance of plants [156]. Essential oils have exhibited greater antimicrobial activity due to a synergistic effect with some active compounds rather than single compound. They have been showing to possess antibacterial activity against *Escherichia coli*, *Enterococcus faecalis*, *Staphylococcus aureus*, *Salmonella* sp., *Vibrio parahaemolyticus*, and *Helicobacter pylori*. They also showed varying degrees of antimicrobial activity against various pathogenic fungi [156,158,159,160,161]. Although antibacterial activity of terpenes remains challenging due to their poor solubility, terpenes show a strong activity especially against Gram-positive bacteria [123,162,163]. Several terpenoid derivatives, such as diterpenoids, also exhibit antimicrobial activity against bacterial fungi, viruses and protozoa with the emphasis on *Mycobacterium tuberculosis* [164]. The antimicrobial mechanisms of terpenes are closely related to their lipophilic features which facilitate their penetration into the microbial cell wall [165]. Monoterpenes preferentially impact the structures of the membrane by increasing its fluidity and permeability, altering the topology of its proteins, and making disturbances across the respiration chain [156]. Moreover, the mechanism of action of terpenoids is not fully understood but is speculated to involve membrane disruption by the lipophilic compounds, disruption of the protein motive force, and coagulation of cell contents [45,166].

## 6. Interaction between Medicinal Plant Extracts and Conventional Antibiotics

There is already evidence for the enhancement of the activity of conventional antibiotics when acting synergistically with plant-derived compounds. The combination of β-lactams with α-mangostin isolated from mangosteen fruit, substantially increase the efficacy of the therapy in β-lactam resistant bacterial strains. It is likely that the mangosteen- derived compounds of those combinations may inhibit the bacterial β-lactamase enzyme, thus reactivating the antibiotic [167]. Several in vitro studies have reported the use of plant extracts in combination with antibiotics with a significant reduction in the minimum inhibitory concentration (MIC) of the antibiotics against some resistant strains. The curative effect of plant extracts in this combination studies has been variably referred to as resistance-modifying activity (RMA) [168,169]. It must be emphasized that plant extract-antibiotics interaction depends on several factors including pharmacokinetics and pharmacodynamics, since combination confirmed in vitro may not have the same effect on humans [170]. Pharmacokinetic interactions occur mainly by increasing the permeability of antibiotics to the bacterial cell membrane or by inhibiting or inducing antibiotic-metabolizing enzymes and transporters, which adversely affect absorption, distribution, metabolism, and excretion of concurrently administered antibiotics. Pharmacodynamic plant extract-antibiotics interactions such as synergism, additive, and antagonist effects are also known to occur [171]. Numerous studies on the interactions between plant extracts and antibiotics can be found in the literature. However, we have compiled some studies summarized below.

*Camellia sinensis* dried leaves extract, together with nalidixic acid, reflected the inhibition of *Salmonella Typhi*. With this combination (C_extract_ = 0.62 mg/mL), nalidixic acid presented a MIC value that was 8-fold lower (32 μg/mL) than when used alone (256 μg/mL) [172]. Moreover, pyridine isolated from *Jatropha elliptica* by bioassay- guided fractionation, at a concentration of 75 μg/mL, was shown to increase by 4-fold the activity of ciprofloxacin and norfloxacin against Nor A expressing *Staphylococcus aureus* when tested at sub-inhibitory concentrations [173]. Isoflavones isolated from the plant *Lupinus argentens*, potentiate the activity of the natural plant antibiotic berberine and the synthetic fluoroquinolone antibiotic norfloxacin. The isoflavone allows a greater concentration of berberine to accumulate in *Staphylococcus aureus* cells by inhibiting the EP mechanism [174]. A study reported that carsonic acid isolated from *Rosmarinus officinalis* L. potentiated the activity of erythromycin [175]. That study determined that the increased erythromycin activity was due to an inhibition of the MDR EP’s by carbonic acid. Similarly, reserpine, a plant alkaloid, isolated from the *Rauwolfia vonitona*. Afzelalso demonstrated effective EP inhibition activity against the bacterial MDR EP, which mediates tetracycline efflux in *Bacillus subtilis* [176]. Fungi have also been evaluated for synergism between plant extracts and antifungals. Synergism has been reported between ketoconazole and *Agastache rugosa* essential oil against *Blastischizomyces capitatus* and between *Pelargonium graveolens* essential oil and amphotericin B *plus* ketoconazole on strains of *Aspergillus* sp. [177,178]. Furthermore, metronidazole showed potentiation of its antifungal effect when combined with *Eugenia Jambolana* L. [179].

Plant extract-antibiotic combinations not only enhance the antimicrobial effect but also can act as resistance modifying/modulating agents. A study reported that *Salvia* spp. and *Martiaria recutita* had synergistic effects with oxacillin, greatly enhancing its efficacy. The authors postulated that it was due to damage to the cytoplasmic membrane of the resistant bacteria and loss of intracellular components [180]. Many medicinal plants acting as MDR EP inhibitors become significant tools when used in combination with some previously ineffective resistance-prone antibiotics. For instance, synergistic activities have been reported for several plant tannins-conventional antibiotic combinations against both resistant and sensitive strains of *Acinetobacter baylyi* [181].

Most studies on the interaction between plant extracts and antibiotics have been focused on the identification and isolation of potential resistance modifiers from medicinal plants. However, it is likely that such combinations could produce antagonistic interactions that many studies have considered irrelevant and thus ignored. However, elucidating synergism and antagonism between plant extracts and antimicrobial drugs is very important. Typical examples are as follows: Synergism assays between terpenes and penicillin against MRSA and *Escherichia coli* revealed a synergistic effect produced by the interaction between carvone and penicillin whereas an antagonistic effect between thymol and penicillin was detected against MRSA strains [182]. Ampicillin, cephalothin, and tetracycline presented synergistic interactions with some essential oils whereas gentamycin mostly had antagonistic interactions [183]. Four essential oils in combination with ciprofloxacin against *Staphylococcus aureus* and *Klebsiella pneumoniae* and with amphotericin B against *Candida albicans* strains revealed synergism or antagonism depending on the type of essential oil and the concentration assayed [184].

## 7. Challenges Surrounding Medicinal Plant Antimicrobial Activity

### 7.1. Use of Antimicrobials from Medicinal Plant Extracts

Antimicrobials of medicinal plant extracts are natural, safer than synthetic alternatives, available in local communities, cheaper to purchase, ease of administration, and they can offer profound therapeutic benefits and more affordable treatment [185,186]. Also, medicinal plant extracts may be a useful alternative treatment in case of numerous side effects and drug resistance [187,188].

The current percentage of approved antibacterial compounds from medicinal plants does not accurately reflect the potential of these compounds for future applications as antimicrobial therapies. Indeed, there are some inherent challenges regarding the use of plant natural extracts as antimicrobial pharmaceuticals:

- Recent studies have shown that medicinal plant compounds should be used with caution in the absence of accurate evidence of their effectiveness [189]. Well-controlled, double-blind toxicological and clinical studies to prove their efficacy and safety are rare [190].
- The use of medicinal plants has been associated with the adulteration of valuable compounds, poor cultivation and collection procedures, lack of standardization during preparation, poor storage conditions, ultimately affecting the process of development of new antimicrobials [191]. Factors such as the season of harvest, region of cultivation, plant parts used, and type of processing can affect the levels and mechanisms of various compounds in extracts. Therefore, the comparison of the different literature data for the plant extracts antimicrobial activity may be problematic due to the composition of plant extracts varying according to local climate and environmental conditions [82]. In particular, rainfall and humidity are versatile in different geographical locations, which could change the composition and production of the compounds when the same species medicinal plant is growing in different geographical locations. Moreover, global changing of climate is another challenge modulating the weather condition and therefore jeopardizing the compounds composition and production, even in the same geographical locations.
- It is difficult for scientists to map all the complex interactions that might be taken place between all the various compounds found in a medicinal plant. The detailed knowledge of the plant extracts composition is another inherent difficulty since these extracts contain many components and hence are difficult to interpret. The isolation of single compounds with the desired antimicrobial activity can be time- consuming and possibly requires a large amount of plant material. Rediscovery of the same compounds from different sources also presents problems. Thus, standardization, stability, and quality control are feasible, but not easy. However, the prospect to study a high quantity of unexplored compounds may contribute to the renovated attention on medicinal plants [192].
- Synergism among compounds in a complex mixture presents unique difficulties as the technology to study multiple compounds acting on potentially multiple biological targets has not yet been fully developed.
- Making arrangements for access to medicinal plant species can sometimes be difficult, especially in an international setting. Regulations concerning plant collation and plant export/import differ depending on where the research is being conducted [193].

### 7.2. Innovative Methods for the Preparation of Medicinal Plant Extracts Chemical Compounds

**Figure 2:**
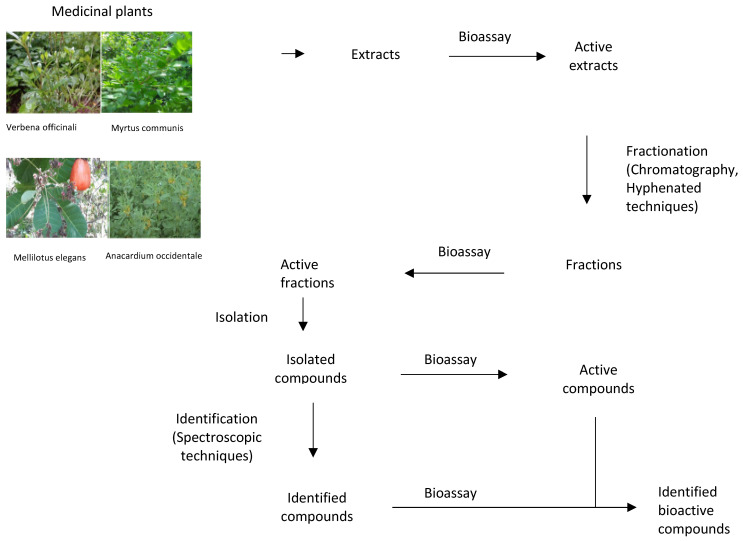
The process of discovering bioactive compounds.

Extraction involves the separation of compounds of plant tissues that are medicinally active from those which are inert by using suitable solvents and appropriate extraction methods (Figure 2). Large numbers of active compounds have been isolated successfully. However, the rate of success and the authenticity of these findings depends on the accuracy in the selection of solvents, selection, and proper execution of extract methods, fractionation, and identification techniques. Thus, researchers need to specialize in standardized solvent systems and extraction methods to attenuate the variation of antimicrobial susceptibility test (AST) results. The choice of solvent depends on the kind of the plant, a part of plant to be extracted, nature of the bioactive compounds, and intended use of the extract. If the aim is antimicrobial compounds screening, the solvent should not inhibit the bioassay procedure [194,195]. Nearby all identified antimicrobial compounds from plants are aromatic or saturated organic compounds and are mostly obtained through initial ethanol or methanol extraction [196,197]. Variations exist in extraction methods based on length of the extraction period, the solvent used, particle size, solvent to sample ratio, temperature, and pH [198,199,200,201]. The suitability of extract methods must be considered and well examined to assure that any bioactive compounds are not lost, distorted, or destroyed during the whole extraction process [202]. The extract obtained may be ready for use or it may be subjected to fractionation and identification to isolate different compounds [203]. Fractionation, a process of separation of plant extracts into various fractions, is based on standardized analytical techniques mainly focusing on the use of chromatography, and hyphenated techniques [203,204]. Identification comprises detection of functional group, presence of multiple bonds and rings, hydrogen, and carbon arrangement as well as full structural elucidation [205]. The methods used include established spectrophotometric techniques [203]. The lack of standard methods to evaluate the antimicrobial activity of medicinal plants is a major challenge. For instance, the agar diffusion assay is not appropriate for the quantitative analysis of medicinal plant extracts as non-polar compounds can fail to diffuse and thus leading to false results. Instead, broth microdilution or agar dilution assays should be used for quantifying the antimicrobial activity of medicinal plant extracts [206]. Some modern extraction methods present certain advantages like comparatively reduced organic sample consumption and sample degradation, fewer steps, improved extraction efficiency, extraction kinetics and ease of automation [205,207]. These methods are proving to be more efficient than the conventional methods [208].

### 7.3. Determination of Antimicrobial Efficacy of Medicinal Plant Extracts

A plethora of assessment tests are currently in use for the determination of the potential antimicrobial efficacy of new medicinal plant extracts [209]. These various ASTs could lead to variation in obtained results [20]. Results obtained will be influenced by the scientific criteria used in the selection of the plant material, the solvent and extraction system, the methodology employed, the composition of the growth medium, and the selected microorganisms [210,211]. The recent standard antimicrobial susceptibility testing methods, which could be broadly categorized into diffusion and dilution methods, might not be exactly applicable to plant extracts and certain modifications must be made [212,213]. The major problem in the diffusion and dilution- based AST is one among the availability of the active principles which may be a function of the solubility of the test compound [214]. Diffusion methods are the qualitative techniques of these methods and give an idea of the presence or absence of antimicrobial substances. Due to its simplicity and ease of performance, diffusion tests were widely adopted by many investigations, but the lack of standardization resulted in unreliable and non-reproducible results [213]. Dilution methods are considered quantitative assays used to determine the MIC or minimum bactericidal concentration (MBC) of antimicrobial agents [215,216]. These methods offer certain advantages over diffusion techniques which include enhanced sensitivity for smaller extract volumes, quantitative analysis, and the ability to differentiate bacteriostatic and bactericidal effects of the extracts [217]. In the broth microdilution method, the assays are performed using small volumes of test antimicrobial and allow bacteria to be tested quite rapidly. The major disadvantage of this method is extensive manual handling of antimicrobial agent solutions, thus increasing the likelihood of errors in solution preparation [218]. Agar dilution offers various advantages like the simultaneous testing of biological isolates, the ability to observe heterogeneous populations or mixed cultures, and the versatility and flexibility in sample selection and concentration range to be tested [218]. Eventually, Etest, an innovative commercial AST combining the principles of both disc diffusion and agar dilution methods, has low variability, gives high reproducible results and its performance has been documented to be equivalent to standard MIC methods [218].

### 7.4. The Challenges of Development New Antimicrobials from Medicinal Plant Extracts

Scientific investigation of new plant extracts is challenging because of their immense complexity and variability [33,219,220]. Indeed, plant extracts may contain hundreds or even thousands of individual compounds in varying abundance and locating the compounds responsible for a given biological effect represents a significant concern [221]. There are several challenges that need to be overcome for the development of new antimicrobials that can face the current spreading of antibiotic resistance:

- The translation of in vitro studies to in vivo experiments and finally to human clinical trials has been the major challenge in the development of new antimicrobials. In vivo research should be carried out for a better understanding of the exact mechanisms of the medicinal plant chemical compounds and whether they can be considered an alternative or supplement to the existing strategies for the treatments of microbial diseases.
- Only medicinal plant extracts that inhibit the growth of microorganisms in low or moderated MIC values should deserve the utmost attention and additional research may be done [24].
- Extracts should also be tested for their MBC, because the bactericidal potential hinders the possibility of antimicrobial resistance.
- The utilization of unique traditional knowledge of medicinal plants medicine has great potential to generate biocompatible solutions and will hasten the discovery of new antimicrobials. This knowledge is important to design efficient and environmentally friendly technologies of fractionation and contribute to the effective exploitation of bioactive plant extracts.
- The method of extraction and in vitro testing should be standardized so that the search for new antimicrobial drugs from medicinal plants could be more systematic, and it will facilitate proper interpretation of results [34].
- Despite the increasing number of compounds isolated from antimicrobial medicinal plants, there are still only relatively few plant-derived drugs in clinical use. This may be because plant compounds often require complex combination effects between components to synergize the activity of the bioactive compound. A number of studies have shown that the overall activity of plant extracts can result from mixtures of compounds with synergistic, additive, and antagonistic activity [33,222,223,224,225]. Therefore, a major concern in the development of antimicrobials from medicinal plants is related to the possibility of synergism or antagonism effects due to the complexity in extracts composition [80,226]. Synergism within and between plant extracts have been extensively reviewed, providing compelling evidence that at least in some cases, the combined effect of plant mixtures is not simply the summation of their individual compounds [33,220,224,227,228,229]. However, it is likely that such combinations could produce antagonism, leading to the cancelation of the therapeutic effect [189]. The classification of combination effects within complex mixtures and the identification of contributing compounds remains a challenging task, particularly when most established tools have been designed to reduce complexity and identify single active compounds of natural products mixtures. Therefore, the use of bioassay-guided or synergy-guided fractionation to predict which compounds/mixtures are responsible for a specific activity is of extreme importance [225].
- Recent developments in metabolomics may play a key role in the identification and effective application of new occurring natural antimicrobials. Statistical modeling used to predict and correlate the metabolomic profile of extracts and their bioactivity has gained much attention [227]. However, there is a lack of *consensus* in the field about which reference models are best for defining combination effects, making the interpretation of studies challenging. Recent models using the specific mean equation [230] and the zero-interaction potency model [231] represent newly developed and robust reference models that may permit improved identification combination effects. There are also several emerging technologies, such as nanotechnology and bio-adhesive technology and materials, namely hydrogel formulations and active packaging materials which can enhance the effectiveness of plant antimicrobial compounds.
- The development of antimicrobials for oral therapy requires the application of methodologies that consider the effect of digestion on the bioactivity of the extracts [232]. However, even using sophisticated in vitro digestion models, it is still impossible to fully mimic the overall digestive parameters in vivo.
- Studies concerning the toxicity of the most promising medicinal plant extracts are major challenge for their use as antimicrobials [233,234]. Most extracts have not been evaluated by the U.S. Food and Drug Administration [235]. Therefore, the lack of official information regarding the actual toxicity of many extracts is concerning, since the adverse effects caused by the misuse of medicinal plants are characterized as a public health problem [236]. Reasons for plant extracts toxicity are improper identification/authentication and improper labeling on standardization. Thus, the extracts should be regulated through official controls and rigorous manufacturing standards.
- Another challenge in the development of antimicrobials from the medicinal plants is related to poor financial support for research and therefore lack of high- quality studies on the comprehension of structure-activity relationship with individual compounds [76].

Despite challenges, there is great demand for the development of new antimicrobials from traditional medicinal plants. The need for new, effective, less expensive, and safer antimicrobials has become a paramount issue for overcoming the above- mentioned challenges, including antimicrobial resistance.

### 7.5. Enhancement of the Antimicrobial Activity of Medicinal Plant Extracts

Efforts have been made to control the content of bioactive compounds. Controlled growth systems may enhance potency, reduce toxin levels, and increase the predictability of extracts. Additionally, traditional, and biotechnological plant-breeding methods can be applied at a genetic level to improve yield and to modify potency or toxicity [237].

Direct manipulation of DNA sequences to alter gene expression in medicinal plants to enhance chemical compound antimicrobial activity has been studied. Genetic transformation of tissue cultures using bacteria to transfer genes into the cultures plant DNA has been employed to improve product output in in vitro systems [235]. Moreover, the increasing production of bioactive compounds through genetic manipulation of medicinal plant biosynthetic pathways presents some challenges. In particular, the metabolic pathways by which bioactive compounds are biosynthesized are mostly poorly understood, and relatively few genes for key enzymatic or regulatory steps are isolated [238].

### 7.6. A Potential ‘ESCAPE’ from Antimicrobial Resistance of ‘ESKAPE’ Pathogens by Exploiting Medicinal Plants

As stated previously, the uncontrolled use of antibiotics, especially in the last four decades, has led to the rise of an unprecedented global health crisis, known as antimicrobial resistance. The truth is that as bacteria have been Earth dwellers for ages, which had already evolved numerous mechanisms to avoid antibiotic attack before the 1930s when the emergence of antibiotics took place, as formerly explained mutations, horizontal gene transfer, toxi-antitoxin systems and mobile genetic elements are amongst the preferred means that microbes finally become ‘superbugs’ as they acquire resistance against multiple, extensively or all agents-Pan drug resistance posing a lethal threat to healthcare settings worldwide [52,239,240].

The Infectious Disease Society of America has already specified a group of bacteria (*Enterococcus faecium*, *Staphylococcus aureus*, *Klebsiella pneumoniae*, *Acinetobacter baumanni*, *Pseudomonas aeruginosa*, and *Enterobacter species*), hereafter referred to as the ESKAPE bacteria, which are especially dangerous due to their virulence and potential antimicrobial resistance [241]. These opportunistic pathogens have incited intense efforts to discover for novel antimicrobial therapies by reinvigorating the antibiotic pipeline aiming to fight against recalcitrant and often lethal infections, especially amongst immunocompromised and critically ill patients.

On the other hand, for a long period of time people mainly from developing countries have relied on traditional medicine to copy with diseases. Up to almost 90% of the population in some countries like Ethiopia take advantage, amongst others, of anti-infective medicinal plants used by traditional healers for the treatment of inflammatory and infectious ailments in primary healthcare system [242]. Plants can synthesize a wide variety of secondary metabolites which may prevent different diseases and inevitably might substitute the use of antibiotics. Therefore, phytochemicals might represent a very promising reservoir of antibiotic adjuvants against infections from ESKAPE pathogens.

Studies up to date have focused on the efficacy of plant-derived extracts on their growth inhibitory action, the prevention of biofilm production (large microbial communities on surfaces resistant to antibiotics) and inhibition of bacterial virulence by targeting quorum sensing (gene regulation depended on cell population density) [243].

The elegant and very enlightening review of Bhatia et al. mentioned 100 plants with meaningful antimicrobial activity against ESKAPE microbes reported over a period of fifteen years (2006–2020) from twelve countries, with the lion’s share originating in India [241]. These plant-derived compounds were prepared in organic solvents or deionised water with the alcoholic extracts presenting the highest antimicrobial activity. The Minimum inhibitory concentration was determined by either the Kirby-Bauer disk diffusion or agar well diffusion methods. Finally, the most common pattern of inhibitory action was against one or two ESKAPE pathogens, but there were a few plant extracts with a broad-spectrum activity namely *Martynia annua*, *Cynodon dactylon* etc. [241].

P-glycoprotein is present in the plasma of ESKAPE pathogens and responsible for the rapid efflux of antibiotics conferring to AMR. Certain plant-derived compounds (*Cynodon dactylon*, *Aloe vera* etc.) have the ability to control the regulation of P-glycoprotein and prevent AMR [244]. The ‘improved intracytoplasmic concentration of antibiotics’ as the study points out is preindicative for the P-glycoprotein activity regulation.

The emergence of ‘superbugs’ encompasses threats towards many of the scientific accomplishments during the last decades. There is a dare need for new strategies to combat AMR and its devastating consequences. The medicinal plants give power to traditional medicine in order to be a new arrow in the antibiotic quiver. A lot of studies from different countries have highlighted the properties of plant extracts for treating ESKAPE-caused infections, but further research is certainly required in order to specify, standardize and unify all the given information into to a commercial antibiotic.

## 8. Study Limitations

Our findings should be interpreted in light of their limitations. The correlation of medicinal plant extracts and antimicrobial activity seems to be significant, however, current knowledge is mainly based on in vitro studies, hence its applicability in the clinical setting remains rather unknown. Compounds that have shown antimicrobial activity in vitro may have little or no effect in vivo. This may be because compounds often require combinational effects between compounds to synergize the activity of bioactive compounds and technology to study this has not yet been fully developed. Indeed, chemometric models are subject to limitations based on the biological assays and reference models used to define biological activity. Additionally, the linear regression models used to predict bioactive compounds are limited given that true linear relationships rarely exist, particularly when assessing complex mixtures with numerous unknown combination effects.

The main limitation of the use of medicinal plants as antimicrobials is the lack of standardization of the treatments. This is one among the explanations for the low credibility regarding the efficacy of medicinal plants. Until recently, the structure-activity relationship and mechanisms of action of bioactive compounds have largely remained elusive. Finding more about the pharmacology of medicinal plant-derived compounds will lead to the standardization of the therapeutic regimens [245].

Clinical trials evaluating the effectiveness of medicinal plant compounds for infectious diseases and the determination of adverse effects are limited [246]. Assessment of the antimicrobial efficacy of pure bioactive compounds requires standardized tests, but more sensitive bioassay techniques are needed to test plant extracts or essential oils [247].

Another important limitation is the reproducibility of the composition of plant extracts. It is known that the same extract may have different properties depending on the supplies. Accurate characterization and authentication of bioactive compounds are necessary to be established quality control procedures [248,249].

The availability of quality plant species is limited to a particular geographical area. Variables ranging from plant species to environmental conditions can influence the availability of quality medicinal plants [250].

## 9. Future Perspectives 

**Figure 3:**
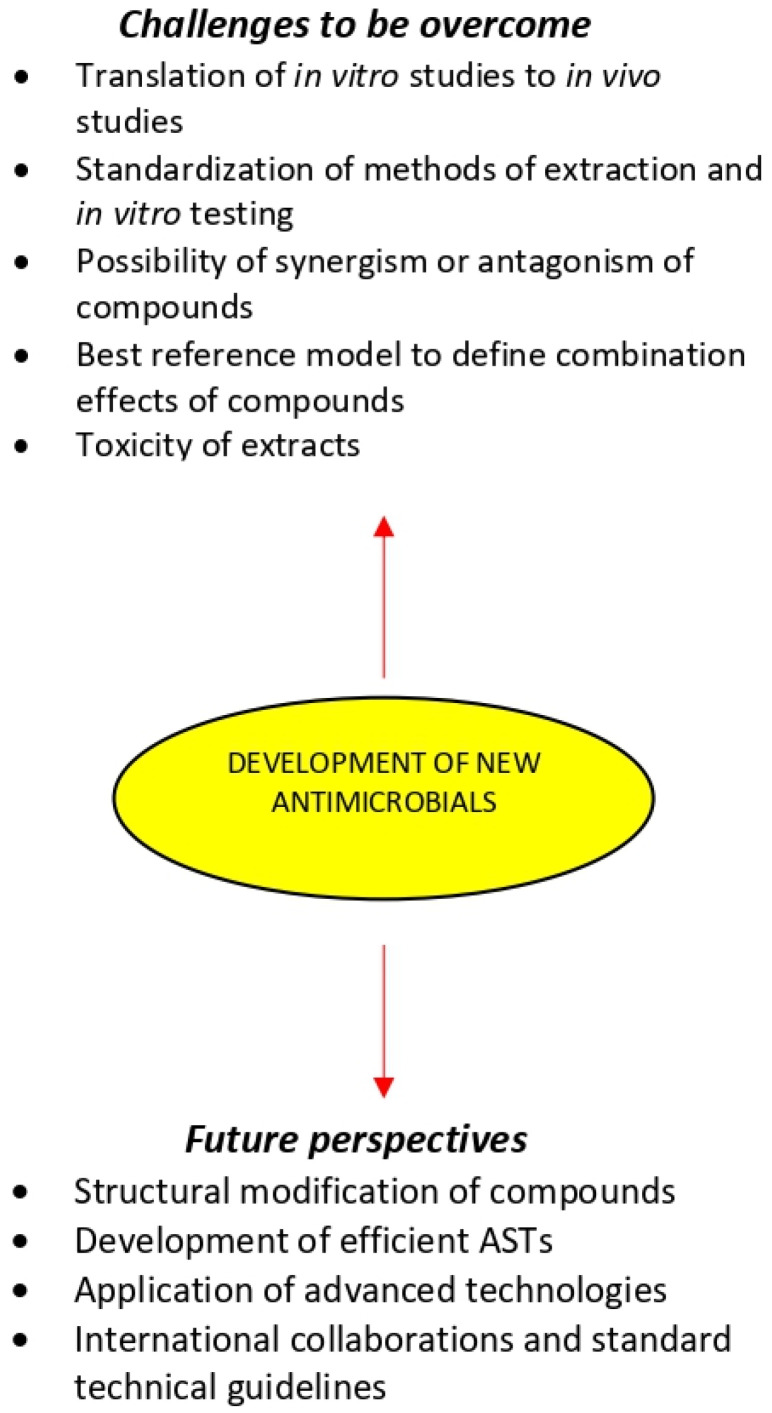
Medicinal plants in the development of new antimicrobials.

Medicinal plants are an underexploited source of bioactive compounds and only a small percentage of their properties have been investigated. As many medicinal plants still remain unexplored, there are important natural resources for the discovery of novel resistance modifying compounds that could become useful therapeutic tools [251]. However, a large part of complex natural compound mixtures awaits chemical investigation representing a resource with considerable potential for further scientific exploration (Figure 3). This could in the future be followed by in vivo testing in animal models of infection to determine the clinical relevance of such compounds and to establish valid correlation with in vitro efficacy results [207]. Further studies should include structural modifications of compounds to improve pharmacokinetics and pharmacodynamics and structure-activity relationship analyses. Synergistic interactions within medicinal plant extracts and between compounds and antibiotics should be further studies to unveil the mechanism beyond the antimicrobial activity of these compounds and then discover multiple pathways to be targeted. However, the interactions between medicinal plant extract and antimicrobial agents can be either favorable such as synergism, or harmful, as in antagonism. Therefore, further studies are required, especially in vivo studies and research on the toxicity of these products to be recognized as a biomedical agent.

The development of efficient ASTs offering advantages over conventional methods for the extraction, isolation, and analysis of bioactive compounds is likely to play an important role in improving the quality of plant antimicrobials [207]. Further studies should be conducted for MIC determination of medicinal plant extracts in order to get comparable results to currently used antibiotics. Already established in vitro ASTs need further fortification by the development of validated in vivo ASTs. Future improvements in the efficiency of ASTs may not entirely rely on developing even more complex techniques, but on implementing best practice throughout all stages of the production and supply of medicinal plant medicines.

Advanced techniques of biotechnological, genomics, proteomics, and metabolomics are nowadays applied to medicinal plant research and contribute to the advancement of alternative natural antimicrobials. Integrated technologies capable of completing the identification of active mixture compounds, characterization of the nature of their interaction, and elucidation of their potential mechanisms of action simultaneously remain to be developed. The confluence of these assessment technologies with advancements in instrument automation will offer remarkable possibilities to exploit the chemical diversity of medicinal plant bioactive compounds in the quest for new antimicrobial drugs.

Increasing consumer demand for effective and safe medicinal plant products means that quantitative data on the activity and ingenious screening programs are required. Scientific organizations are required to develop standard technical guidelines for the analysis of medicinal plant extracts, to be ready to measure and compare results of the growing research in this field. International collaborations between resource-rich institutions with partners in biodiversity- rich areas of the world share expertise and lead to benefits for research teams, government bodies, and community partners. Such collaborations can result in research training opportunities for students and faculty [252].

## 10. Conclusions

Medicinal plant antimicrobial activity is a new hope to combat the dangerous threats posed by increasing evidence of antimicrobial resistance. Therefore, there is an urgent need to identify and isolate new bioactive compounds from medicinal plants, which have yet to be adequately explored. The large diversity of these compounds has proved to have therapeutic potentials as antimicrobials and as antimicrobial resistance modifiers.

The potential use of new bioactive compounds is still challenging. It is essential to emphasize that extensive in vitro and in vivo tests must be conducted to assure the selection of active and nontoxic antimicrobial plant-derived compounds. It is also a major challenge to exploit the potential synergistic or antagonistic effects of compounds within and between medicinal plant extracts.

As biotechnology advances, it is obvious that we will be able to search further into the chemical composition of medicinal plants and develop more sophisticated techniques for the extraction, fractionation, and identification of bioactive compounds which are characterized by diverse chemical structures and mechanisms of action. It would be advantageous to standardize methods of extraction and in vitro testing in order that the search might be more systematic, and interpretation of results would be facilitated. Additionally, reference models have yet to be employed in studying plant extract mixtures and future studies will reveal their applicability for this approach.

Studies on the mechanisms of action, interactions with antibiotics or other medicinal plants or compounds, and the pharmacokinetic and pharmacodynamic profile of the extracts should be given high priority.

It is expected that this review and the main challenges that were identified in this field would be helpful in the use of more efficient, successful, and straightforward methods to get to the use of new therapeutic medicinal plants more quickly against microbes.

## Figures and Tables

**Table 1:** Some important medicinal plant extracts compounds with antimicrobial activity.

Plant Sources	Class of Compound	Compound	Mechanisms	Susceptible Microorganism	References
*Rauwolfia serpentine*	Alkaloid	Reserpine	EP inhibitor	*Staphylococcus* sp., *Streptococcus* sp., *Micrococcus* sp.,	[92]
*Piper nigrum*		Piperine	EP inhibitor	MRSA, *Staphylococcus aureus*	[93]
		Conessine	EP inhibitor	*Pseudomonas aeruginosa*	[94]
		Berberine	Protein and DNA synthesis inhibitor	*Escherichia coli*, *Candida albicans*	[95]
*Berberis vulgaris*		Tomatidine	ATP synthetase inhibitor	*Listeria, Bacillus* *Staphylococcus spp.*	[96]
	Phenolic compound/polyphenols	Rhamentin	EP inhibitor	*Staphylococcus aureus*	[97]
*Camellia sinensis*		Epigallocatechin gallate	Beta-ketoacyl-reductase	*Escherichia coli*	[98]
		Chebulinic acid	DNA gyrase	*Mycobactrium tuberculosis*	[99]
		3-p-Trans-coumaroyl-2-hydroxyquinic acid	Cell membrane damage	*Staphylococcus aureus*	[100]
*Cedrus deodara*		Apigenin	d-Alanine:d-alanine ligase	*Helicobacter pylori*, *Escherichia coli*	[101]
*Allium sativum*	Sulfur-containing compounds	Allicin	Protein and DNA synthesis inhibitor	*Staphylococcus epidermidis*, *Pseudomonas aeruginosa*, *Streptococcus agalactiae*	[102]
*Rubus ulmifolius*		Ajoene	Sulphydryl-depe endent enzyme inhibitor	*Cambylobacter jejuni*, *Streptococcus*, *Staphylococcus* *Escherichia coli*	[88]
		Sulforaphane	Destruction of bacterial membrane, Protein and DNA synthesis inhbitor, ATP synthase inhibitor	*Escherichia coli*	[103]
		Alyssin		*Helicobacter pylori*	[83]
*Raphanus sativus*		Allyl isothiocyanate Benzyl isothiocyanate Phenethyl isothiocyanate		*Bacillus subtilis*, *Staphylococcus aureus*, *Staphylococcus epidermidis*, *Enterococcus faecalis*, *Salmonella typhimurium*, *Enterobacter cloacae*, *Escherichia coli*	[104]
*Ferulago campestris*	Coumarin	Aegelinol	DNA gyrase inhibitor	*Salmonella enterica serovar Typhi*, *Enterobacter aerogenes*, *Enterobacter cloacae*, *Staphylococcus aureus*	[105]
		Agasyllin	DNA gyrase inhibitor	*Salmonella enterica serovar Typhi*, *Enterobacter aerogenes*, *Enterobacter cloacae*, *Staphylococcus aureus*, *Helicobacter pylori*	[105]
*Prangos hulusii*		4′-senecioiloxyosthol	DNA gyrase inhibitor	*Bacillus subtilis*	[106]
		Osthole	DNA gyrase inhibitor	*Bacillus subtilis*, *Staphylococcus aureus*, *Klebsiella pneumoniae*, MRSA	[106,107]
*Mesua ferrea*		Bergamottin epoxide	EB inhibitor	MRSA	[108]
*Thymus vulgaris*	Terpene	Furnesol	Cell membrane disturbance	*Staphylococcus aureus*	[109]
		(4R)-carbone	Cell membrane disturbance	*Cambylobacter jejuni*, *Enterococcus faecalis*, *Escherichia coli*	[110]
		Thymol	Cell membrane (H+)-ATPase inhibition, Cell membrane disturbance, EP inhibitor	*Candida albicans*, *Candida glabrata*, *Candida crusei*, *Escherichia coli*, *Staphylococcus aureus*, *Pseudomonas aeruginosa*, *Aspergillus niger*, *Aspergillus flavus*, *Fusarium oxysporum*	[111]
		Carvacrol	Cell membrane disturbance, EP inhibitor	*Escherichia coli*, *Enterobacter aerogenes*, *Staphylococcus aureus*, *Pseudomonas aeruginosa*, *Salmonella typhimarium.* *Aspergillus niger*, *Aspergillus fumigatus*, *Epadosporium spp.*, *Rhizopus oryzae*	[112]
		Cinnamaldehyde	Cell membrane disturbance	*Helicobacter pylori*	[113]
